# Limitations of the 3-(4,5-dimethylthiazol-2-yl)-2,5-diphenyl-2H-tetrazolium bromide (MTT) assay when compared to three commonly used cell enumeration assays

**DOI:** 10.1186/s13104-015-1000-8

**Published:** 2015-02-20

**Authors:** Alet van Tonder, Annie M Joubert, A Duncan Cromarty

**Affiliations:** Department of Pharmacology, University of Pretoria, Pretoria, South Africa; Department of Physiology, University of Pretoria, Pretoria, South Africa

**Keywords:** MTT assay, Neutral red uptake assay, Resazurin reduction assay, Sulforhodamine B assay, 3-bromopyruvate, 2-deoxyglucose, Lonidamine, Assay interference

## Abstract

**Background:**

The tetrazolium-based MTT assay has long been regarded as the gold standard of cytotoxicity assays as it is highly sensitive and has been miniaturised for use as a high-throughput screening assay. However, various reports refer to interference by different test compounds, including the glycolysis inhibitor 3-bromopyruvate, with the conversion of the dye to coloured formazan crystals. This study assessed the linear range and reproducibility of three commonly used cell enumeration assays; the neutral red uptake (NRU), resazurin reduction (RES) and sulforhodamine B (SRB) assays, in comparison to the MTT assay. Interference between the MTT assay and three glycolysis inhibitors, 2-deoxyglucose, 3-bromopyruvate and lonidamine, was investigated.

**Results:**

Data indicate that the NRU, RES and SRB assays showed the smallest variability across the linear range, while the largest variation was observed for the MTT assay. This implies that these assays would more accurately detect small changes in cell number than the MTT assay. The SRB assay provided the most reproducible results as indicated by the coefficient of determination after a limited number of experiments. The SRB assay also produced the lowest variance in the derived 50% inhibitory concentration (IC_50_), while IC_50_ concentrations of 3-bromopyruvate could not be detected using either the MTT or RES assays after 24 hours incubation. Interference in the MTT assay was observed for all three tested glycolysis inhibitors in a cell-free environment. No interferences were observed for the NRU, SRB or RES assays.

**Conclusions:**

This study demonstrated that the MTT assay was not the best assay in a number of parameters that must be considered when a cell enumeration assay is selected: the MTT assay was less accurate in detecting changes in cell number as indicated by the variation observed in the linear range, had the highest variation when the IC_50_ concentrations of the glycolysis inhibitors were determined, and interference between the MTT assay and all the glycolysis inhibitors tested were observed. The SRB assay performed best overall considering all of the parameters, suggesting that it is the most suitable assay for use in preclinical screening of novel therapeutic compounds with oxido-reductive potential.

**Electronic supplementary material:**

The online version of this article (doi:10.1186/s13104-015-1000-8) contains supplementary material, which is available to authorized users.

## Background

Developing a new therapeutic agent from conception, initial testing in the laboratory to availability on the market is a long and costly process: it is estimated that approximately U$868 million is spent on the successful approval of a single drug [1]. Preclinical testing is vitally important to eliminate unsuitable candidates before the expense of clinical research is incurred. The use of cell culture for the initial preclinical screening of potential therapeutic compounds has become commonplace as cultured cells can be selected to represent the disease of interest or its associated biochemical anomalies [2]. It is of vital importance to obtain accurate, reliable results from the *in vitro* cytotoxicity assays employed in the initial stages of preclinical research as this data may influence the success of a drug candidate to proceed into the development process.

The 3-(4,5-dimethylthiazol-2-yl)-2,5-diphenyl-2H-tetrazolium bromide (MTT) assay has become the gold standard for determination of cell viability and proliferation since its development by Mosmann in the 1980′s [3]. This assay measures cell viability in terms of reductive activity as enzymatic conversion of the tetrazolium compound to water insoluble formazan crystals by dehydrogenases occurring in the mitochondria of living cells although reducing agents and enzymes located in other organelles such as the endoplasmic reticulum are also involved [4,5]. The increased sensitivity of the assay and its potential as a miniaturised high-throughput assay made it a breakthrough in cell enumeration technology by replacing the radioactive isotope based ^3^H-thymidine incorporation assay. Initially, the method involved no wash steps, but called for the solubilisation of the formazan crystals in acid-isopropanol, a time-consuming procedure [3]. However several modifications, including the addition of DMF to solubilise the formazan in aqueous medium [6] or removing excess dye with gentle aspiration and washing with PBS followed by solubilising the formazan crystals in DMSO [7] improved the simplicity and sensitivity of this assay. Several tetrazolium-based assays, such as the XTT [8], MTS [9] and WST [10] assays, in which water soluble formazan products are generated, eliminating the need for washing and solvent solubilisation steps, have been developed but have not replaced the well-established MTT assay.

A recent report indicated that certain glycolysis inhibitors, such as 3-bromopyruvate, interferes with the MTS assay [11]. A more thorough literature review revealed that several tetrazolium-based assays, such as the MTT and MTS assays, show interactions with many phytochemicals demonstrating intrinsic reductive potential including antioxidants [12,13] and polyphenols [14], compounds generating superoxide such as nano titanium dioxide [15], and corrosion products of certain metal alloys [16]. Furthermore, the dependence of the MTT assay on metabolic function can confound results as a direct correlation between the glucose concentration of the cell culture medium and the reductive rate of MTT has also been observed [17]. Increased reduction of the MTT dye has been reported in the presence of liver fractions indicating the reductive potential of various hepatic cytosolic and microsomal enzymes [18].

In this study the MTT assay, considered by many to be the ‘gold standard’, was compared to three commonly used cell enumeration assays: the neutral red uptake assay (NRU), resazurin reduction assay (RES) and the sulforhodamine B assay (SRB). The tetrazolium-based MTT assay relies mainly on enzymatic conversion of the dye to formazan crystals which occurs in numerous organelles including the mitochondria and endoplasmic reticulum [5,6] however it has become evident that many endogenous and exogenous compounds can also catalyse this chemical change. The conversion of resazurin to fluorescent resorufin occurs mostly in the mitochondria and the quantity of resorufin generated can therefore be used as indicator of metabolic activity [19]. The neutral red uptake assay relies on the intracellular accumulation of the dye in cellular lysosomes via active transport [20]. The sulforhodamine B assay in contrast measures total cellular protein content and does not rely on cell functionality [21,22]. At present the SRB assay is the preferred high-throughput assay of the National Cancer Institute (NCI) in the USA and is the assay used in the NCI’s lead compound screening programme [21-23].

When considering a cell enumeration assay a number of variables must be taken into account including potential interferences, linearity, sensitivity and reproducibility of the assay. Assays used in the initial screening of potential anticancer compounds must be sufficiently sensitive to detect small differences in cell number, yet robust enough to generate reproducible results under various controlled experimental conditions. Further advantages for an assay are a simple experimental procedure so that reliable data is obtained from the first experiment and the potential for automation. These characteristics would ensure that *in vitro* cytotoxicity data can be obtained in a time- and cost-effective manner.

Based on these reports the potential for interference between the MTT assay and two hexokinase inhibitors as well as lonidamine which all inhibit the glycolysis pathway were investigated. The hexokinase inhibitors, 2-deoxyglucose (2DG) and 3-bromopyruvate (3-BrPA), abrogate the conversion of glucose to glucose-6-phosphate by hexokinases [24]. In contrast, exposure to lonidamine (LON) results in damage to the mitochondria and ultimately the cessation of glycolysis [25]. Aerobic glycolysis is the favoured energy production pathway of cancerous tissue, a phenomenon known as the Warburg effect [26]. Normal cells do not exhibit high rates of glycolysis under aerobic conditions as glycolysis leads to the generation of only two molecules of ATP per molecule of glucose used, whereas the Krebs cycle produce 36 molecules of ATP for every molecule of glucose consumed [27]. The conversion of glucose to glucose-6-phosphate by hexokinases is the only rate-limiting step in the glycolytic pathway and therefore a very attractive target for potential chemotherapeutic agents [28]. Furthermore, as these inhibitors exert an effect on mitochondrial activity the probability that these glycolysis inhibitors could interfere with cell enumeration assays should be considered before experimental protocols are finalised.

To determine the most reliable and sensitive cell enumeration assay for preliminary screening of potential anticancer agents in the presence of co-administered glycolysis inhibitors prompted an investigation of the linear range, reproducibility, potential interference and cost-effectiveness of four commonly used cell enumeration assays used in cytotoxicity evaluation.

## Methods

### Cell culture

Approval for the use of commercially available cell culture was obtained from the Research Ethics Committee of the University of Pretoria (307–2013).

MDA-MB-231, MCF-7 and MCF-12A cell lines were purchased from the American Type Culture Collection. Tissue culture medium and supplements, dyes, glycolysis inhibitors and dimethyl sulfoxide were obtained from Sigma-Aldrich Chemical Co (St Louis, USA). Phosphate buffered saline (PBS) was procured from BD Biosciences (Sparks, USA) and foetal bovine serum (FCS) from PAA Laboratories (Pasching, Austria).

### Linear range

To determine the linear range of each assay, six cell densities ranging from 50 – 10 000 cells/well were plated into sterile 96-well plates and incubated for 24- or 72 hours in the absence of glycolysis inhibitors before performing one of the four cell enumeration assays.

### Reproducibility

To investigate the reproducibility of each assay, cells were plated into sterile 96-well plates at a concentration of 500 cells/well and allowed to attach for 24 hours before exposure to varying concentrations of 3-bromopyruvate, lonidamine or 2-deoxyglucose. Internal triplicates for each concentration were included in each experiment. After a further 24- or 72 hours incubation period, cell enumeration assays were performed. From the data obtained after four independent experiments the concentration of each inhibitor which inhibits 50% cell viability (IC_50_) as measured by each enumeration assay with triplicate repeats was determined using GraphPad Prism® version 4.0 for Windows (GraphPad Software, San Diego California USA, www.graphpad.com) and the variability of the IC_50_ concentrations was compared.

### Interference

To investigate potential interference of each cell enumeration assay by the glycolysis inhibitors, the assay dyes were diluted as required in PBS until an absorbance reading of approximately 1.2 was obtained in sterile 96-well microtitre plate wells. The glycolysis inhibitors were also diluted in PBS and added to the dyes. After an incubation period of 4 hours, the absorbance was determined spectrophotometrically at the wavelengths used for each assay. Three independent experiments were performed.

### Cell enumeration assays

#### Neutral Red Uptake assay (NRU)

The NRU method was used as previously described [29]. After a 24- or 72 hours incubation period, the 96-well microtitre plate was centrifuged (230 *g* for 10 minutes) and the medium was removed using a multichannel auto pipette. Neutral red stain (100 μl of a 0.2 mg/ml in cell culture medium) was added to each well and the plate was re-incubated at 37°C for 4 hours. Thereafter the plate was washed with PBS that was removed then, allowed to dry for approximately 1 hour and 100 μl of neutral red eluent (EtOH:dH_2_O:acetic acid 50:49:1) added to each well. The microtitre plate was placed on a shaker for 1 hour in order to dissolve the dye. After the neutral red had dissolved, the absorbance of the plate was determined spectrophotometrically at 540 nm with a reference wavelength of 630 nm using an ELX800 UV universal microplate reader (Bio-Tek Instruments Inc., Vermont, USA).

#### MTT assay

The MTT staining method as described by Mosmann [3] was used with minor modifications [7]. After a 24- or 72 hour incubation period, 20 μl of a 5 mg/ml MTT solution was added to each well and the plate was further incubated at 37°C for 4 hours. Thereafter the medium was aspirated and the wells washed with PBS, allowed to dry for approximately 2 hours and 200 μl of DMSO was added to each well. The microtitre plate was placed on a shaker in order to dissolve the dye. After the formazan crystals had dissolved, the absorbance was determined spectrophotometrically at 570 nm using a reference wavelength of 630 nm on an ELX800 UV universal microplate reader (Bio-Tek Instruments Inc., Vermont, USA).

#### Resazurin reduction assay (RES)

This assay was performed colorimetrically as previously described [19]. After a 24- or 72 hours incubation period, the 96-well microtitre plate was centrifuged (230 *g* for 10 minutes) and the medium removed using a multichannel auto pipette. Resazurin stain (100 μl of 0.025 mg/ml in PBS) was added to each well using a multichannel auto pipette and the plate re-incubated at 37°C for 4 hours. Thereafter the absorbance was determined spectrophotometrically at 570 nm using a reference wavelength of 630 nm on an ELX800 UV universal microplate reader (Bio-Tek Instruments Inc., Vermont, USA).

#### Sulforhodamine B assay (SRB)

The assay was done according to the method described by Vichai and Kirtikara [22]. After a 24- or 72 hours incubation period, cells were fixed by adding trichloroacetic acid to a final concentration of 10% trichloroacetic acid to the wells and the plate was incubated at 4°C for 24 hours. Thereafter, the plate was gently washed under flowing tap water and allowed to dry for approximately 1 hour before staining with 100 μl of SRB stain (0.057% in 1% acetic acid). After 30 minutes the plate was washed with 1% acetic acid to remove excess stain. The plate was again allowed to dry for approximately 30 minutes. A volume of 200 μl of TRIS buffer (10 mM, pH 10.5) was added and the plate was placed on a shaker to dissolve the stain for approximately 30 minutes. The absorbance was determined spectrophotometrically at 540 nm using a reference wavelength of 630 nm on an ELX800 UV universal microplate reader (Bio-Tek Instruments Inc., Vermont, USA).

### Interpretation of results

A minimum of three independent experiments with a minimum of eight internal replicates were performed to determine the linearity of each cell enumeration assay. For reproducibility and interference experiments a minimum of three independent experiments with a minimum of three internal replicates were performed. All data was blank adjusted prior to further interpretation. All statistical calculations and generation of graphs were completed using GraphPad Prism® version 4.0 for Windows (GraphPad Software, San Diego California USA, www.graphpad.com).

## Results and discussion

To aid with the discussion, some advantages and disadvantages of each cell enumeration assay are listed in Table 1. Although numerous studies have compared cell enumeration assays, this appears to be the first report comparing the NRU, MTT, RES and SRB *in vitro* assays under the same conditions. This study compared the cell enumeration assays based on linear range, reproducibility and interference with glycolysis inhibitors.

**Table 1:** **A summary of the advantages and disadvantages of the four cell enumeration assays**

	**Advantages**	**Disadvantages**
**Neutral red uptake assay**	1. Cell enumeration independent of enzymatic conversion of dye [30,31]	1. Some reports of test compound interference [32]
	2. Few wash steps involved [30,31]	
**MTT assay**	1. Gold standard for cytotoxicity testing	1. Conversion to formazan crystals depends on metabolic rate and number of mitochondria resulting in many known interferences [4,12-17,33]
	2. Suitable for high-throughput screening and miniaturisation [34]	2. Numerous wash steps required [3,7]
**Resazurin reduction assay**	1. Few wash steps involved [19]	1. Conversion to resorufin depend on enzymatic conversion [18]
	2. Follow-up assays can be performed on same cells as assay is not cytotoxic [32,35]	2. Absorbance-based method less sensitive than fluorescence-based method
**Sulforhodamine B assay**	1. Cell enumeration dependent on protein content thus no test compound interference [21,22]	1. Numerous wash steps involved, but fixation required [22]
	2. Highly reproducible	2. Less sensitive with non-adherent cells

### Linear range

**Figure 1:**
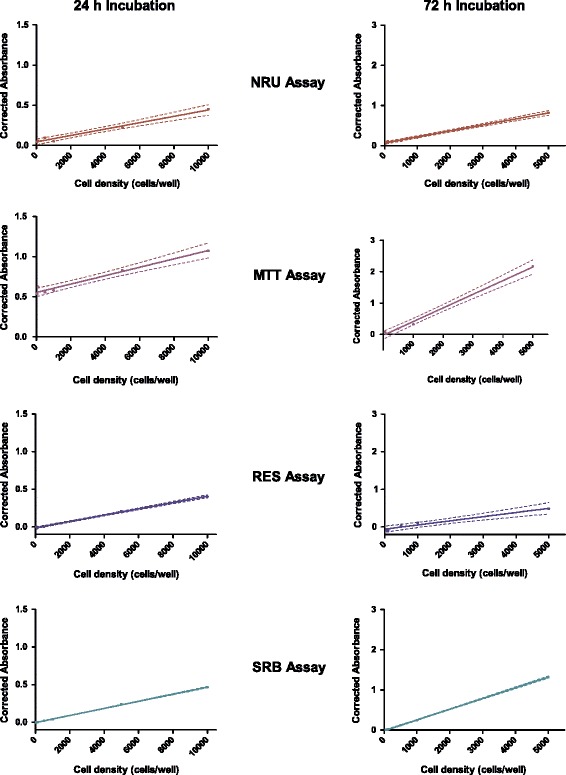
**The linear range of the four cell enumeration assays using MDA-MB-231 cells.** Four different cell enumeration assays were performed after 24 and 72 h incubation periods at six increasing starting cell densities. The solid line represents the linear least squares fit of the data. The dashed lines represent the 95% confidence bands. Graphs for 24 h incubation period depicts cell density up to 10 000 cells/well and 72 h incubation period depicts cell density up to 5 000 cells/well (n = 4).

The linear range of each assay using a range of cell concentrations over 24- and 72 hours incubation without any drug exposure revealed comparable results for the three different cell lines used. The results obtained for the MDA-MB-231 cell line with the 95% confidence bands indicated, indicating the percentage of the data which can be explained by regression analysis, as well as the absorbance obtained for 50 and 100 cells/well are shown in Figure 1. After both incubation periods, the SRB assay shows the lowest variability as seen with the narrow 95% confidence bands, indicating that this assay is potentially the most accurate and sensitive to changes in cell number. The largest variation in linear range was observed for the MTT assay, indicating that operator experience with the assay as well as numerous other experimental parameters may influence the results obtained.

The high background absorbance values obtained from the MTT assay at the lowest seeding densities after 24 hours suggest that this assay is less sensitive at cell numbers below 1000 cells/well. The RES and SRB assays were able to detect differences in cell number as low as 500 cells/well accurately. Even though low seeding densities are not often employed, the cytotoxic effects of novel therapeutic agents may lead to such diminished cell numbers. For the screening of novel therapeutic agents the SRB assay seems superior to the MTT assay due to greater sensitivity.

It is evident that the absorbance signal is directly proportional to the cell number, which is expected to increase with incubation time. This is most clearly seen after 72 h incubation where the MTT assay was performed (Figure 1). Some reports have however indicated that the quantity of formazan crystals produced is not solely dependent on cell number [33,36]. The MTT assay relies on the conversion of the tetrazolium dye to formazan by mainly mitochondrial succinic dehydrogenases, although cytosolic enzymes such as nicotinamide adenine dinucleotide (NADH) reductase and flavin oxidase may also be involved [4]. The rate of the conversion to the formazan has been shown to be linked to the metabolic activity of the cell and therefore decreased intracellular glucose concentrations may result in a decrease in the amount of formazan crystals produced [36]. Another factor reported to influence the conversion to formazan is the number of mitochondria present in the cell. Accordingly, larger cells with more mitochondria have a higher rate of tetrazolium conversion [33]. Defective mitochondria have also been reported to retain the ability to reduce tetrazolium [37]. Taken together these factors may lead to a false estimation of cell number.

The coefficient of determination (r^2^), indicative of how closely the growth curve generated can be fitted with non-linear regression statistics for the cell lines and incubation periods tested, is shown in Table 2. An r^2^ value of less than 0.9 was calculated for the MTT and NRU assays after 24 h incubation using the MCF-7 and MCF-12A cell lines respectively while an r^2^ value of less than 0.9 was calculated for the MTT and RES assays after 72 h incubation. The SRB assay was the only assay for which the non-linear regression statistics could be fitted closely to experimental data obtained after both these incubation times tested, suggesting that the most reliable results will be obtained using the SRB assay.

**Table 2:** **The coefficient of determination (r** ^**2**^ **) of the four cytotoxicity assays**

**Cell lines and incubation periods**		**NRU**	**MTT**	**RES**	**SRB**
MCF-7	24 hours incubation	0.901	0.711	0.999	0.992
MDA-MB-231		0.979	0.975	0.998	0.999
MCF-12A		0.846	0.903	0.976	0.999
MCF-7	72 hours incubation	0.994	0.762	0.915	0.999
MDA-MB-231		0.958	0.878	0.843	0.973
MCF-12A		0.986	0.990	0.887	0.985
**Average**		**0.937**	**0.864**	**0.934**	**0.991**

The neutral red, MTT, resazurin reduction and SRB assays were performed after 24 or 72 hour incubation on three cell lines (n = 4).

Data obtained during this study regarding the wide linear range of the SRB assay confirms published results [38]. The highest average coefficient of determination was calculated for the SRB assay indicating greater predictive power of the assay. Of the four assays, the SRB assay is the only true cell enumeration method as it does not rely on a metabolic function or activity of live cells for quantification of cell number [22]. Instead the assay relies on the ability of SRB to bind to basic amino acids under slightly acid conditions, while bound stain is released under strongly basic conditions [22]. This approach eliminates the influence of varying biological parameters, such as increased metabolic rate or cellular mitochondria number on the quantification process. This data indicates that the SRB assay appears to be the most effective in detecting small or large changes in cell number.

Hamid and colleagues who conducted an extensive study comparing the MTT and RES assays, concluded that while both assays are useful as cytotoxicity assays, the RES assay is more suited for HTS as it is more sensitive [34]. In the present study the linear range for the RES assay was slightly wider than that determined for the MTT assay; but more importantly the overall coefficient of determination calculated for the RES assay was better than that of the MTT assay. It should be considered, however, that an absorbance-based version of the RES assay was used in this study and that the sensitivity and selectivity of the assay would be further enhanced using fluorescence detection. In contrast to the MTT assay, the resazurin reduction assay offers the advantage of allowing follow-up assays as the reagents and the dye used in the RES assay do not influence cell viability [35]. Unlike the MTT and other tetrazolium-based assays which abrogate respiration, the RES dye acts as an electron acceptor in the last step in the respiratory chain and does not produce a toxic effect [32]. Unfortunately, FCS has been reported to interfere in the RES assay [19], but this obstacle may be easily overcome by removing cell culture medium at the end of the incubation period and using serum free media as a solvent for the resazurin dye instead of complete cell culture medium.

### Reproducibility

**Figure 2:**
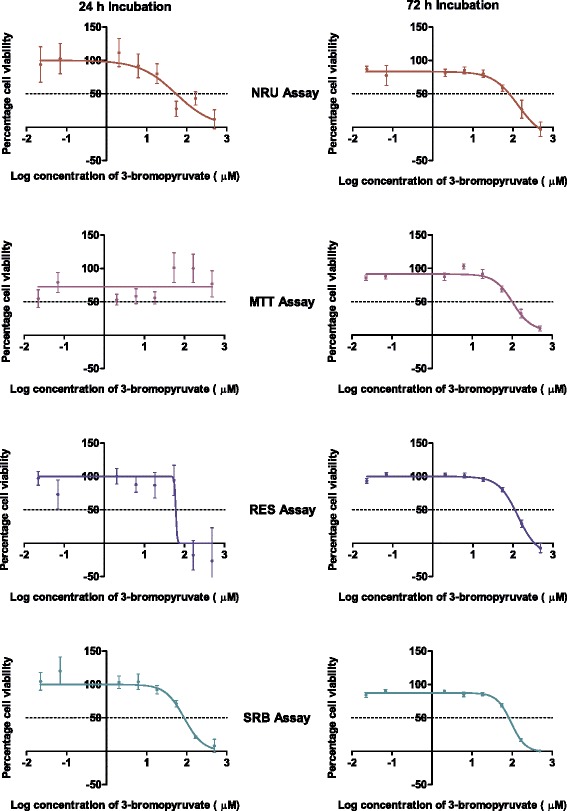
**The effect of 3-bromopyruvate on the growth of MCF-7 cells.** The graphs represent results obtained after 24 and 72 hours incubation as assayed by the four cell enumeration assays. Note that the error bars are smaller in the 72 hour incubation graphs and in many of the data points fall within the symbol (n = 3).

Enhanced reproducibility translates into requiring fewer experimental repeats, and thus less reagent and consumables, to obtain reliable data. The implication regarding costs and experimental time cannot be underestimated. Comparing the reproducibility of the assays obtained in the IC_50_ concentrations of the glycolysis inhibitors calculated after four independent experiments with internal triplicates provides an effective means to rank the assays according to reproducibility. Results for the non-linear regression statistics were used to generate the dose–response curves for each assay on the MCF-7 cell line after 24- and 72 h incubation periods (Figure 2). It appears that the data points for the MTT and SRB assays most closely resemble the dose–response curves suggesting that these assays would produce the most reliable results. After three experimental repeats, the SRB assay showed the lowest overall variability at both the 24- and 72 h time points and for all three cell lines tested (Table 3) implying that fewer experimental repeats would be required with this assay to obtain reliable results.

**Table 3:** **The IC**_**50**_ **concentrations of 3-bromopyruvate calculated for MCF-7 cells after 24 or 72 hour incubation**

	**Cytotoxicity assay**			
	**NRU**	**MTT**	**RES**	**SRB**
24 hour Incubation	54.1 ± 1.6	-	-	86.4 ± 1.2
72 hour incubation	60.9 ± 1.3	98.5 ± 1.1	105.9 ± 1.1	74.4 ± 1.1

Where no values are given these could not be calculated as the assay reported relative cell survival of greater than 50% at the tested concentrations (n = 4).

Results are indicated as mean ± SEM.

The IC_50_ concentrations of the glycolysis inhibitors could only be determined at both incubation time points using the NRU and SRB assays, but not with the MTT assay (Figure 2, Additional file 1) which may indicate a lack of sensitivity of this assay. However, a previous study reported an IC_50_ concentration of 84.6 ± 15.4 μM for 3-BrPA using MCF-7 cells after 16 h incubation using the MTT assay where the formazan crystals were solubilised using an isopropanol solution [39]. After 16 h incubation, only a small percent of the MCF-7 cells would have divided and fewer cells would be available to convert the tetrazolium dye into the formazan product, suggesting that an IC_50_ concentration after 24 h should be possible. However, the difference in the solubilisation solvent used, isopropanol as opposed to DMSO, could influence the results obtained.

### Interference studies

**Figure 3:**
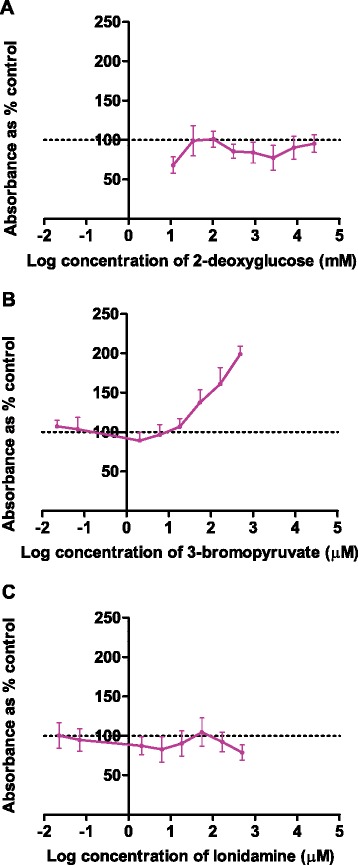
**Interference of three glycolysis inhibitors with the MTT assay in a cell-free system.** After 4 hours incubation with **(A)** 2-deoxyglucose, **(B)** 3-bromopyruvate and **(C)** lonidamine interference was seen for the MTT assay (n = 3).

The potential interference between the glycolysis inhibitors and the dyes used in each of the cell enumeration assays were investigated using cell free assays and the results shown in Figure 3. The absorbance of the tetrazolium dye used in the MTT assay does not remain constant when any of the glycolysis inhibitors are incubated with the dye for 4 h in a cell free system. Interestingly, the absorbance does not demonstrate a consistent dose dependent change in the presence of all of the glycolysis inhibitors: the absorbance increases in a dose-dependent manner when exposed to 3-BrPA, but decreased with 2DG and LON. No interference as measured by colour change was observed for the NRU, SRB or RES assays with any of the tested glycolysis inhibitors (see Additional file 2).

Interference between glycolysis inhibitors and cell enumeration assays has been reported previously [11]. Of the three glycolysis inhibitors selected for this study, 3-bromopyruvate and 2-deoxyglucose inhibit hexokinase, the enzyme responsible for the conversion of glucose to glucose-6-phosphate in the first rate-limiting step of the glycolytic pathway [24]. The glucose analogue 2DG competes for conversion by hexokinase with glucose. The product of this conversion, 2-deoxyglucose-6-phosphate, is not metabolised by downstream hydrolytic enzymes and thus the cells slowly starve [24].

The observed interference between the glycolysis inhibitors and the tetrazolium MTT dye was significant (approximately 2-fold increase in absorbance) and strongly suggests that this assay is not suitable for use with glycolysis inhibitors. This confirms previously published data on the interaction between 3-BrPA and tetrazolium-based assays [11]. Data obtained in this study suggests interference of the MTT assay by 2DG. However, previous studies using 2DG and the MTT assay have been reported without any mention of interference by 2DG [40]. The decreased absorbance noted after incubation of MTT with 2DG and LON cannot be explained based on the available information and the mechanism involved should be investigated further. Interestingly the RES assay, which relies on a similar reduction mechanism as the MTT assay, did not show interference from any of the three glycolysis inhibitors (Additional file 1). The lack of interference observed for the SRB and NRU assays could be explained by the different dye binding targets providing the colour in these assays. To the author’s knowledge no reports on interference with the SRB assay by experimental compounds have been published to date.

## Conclusions

Although generally accepted as the ‘gold standard’, this study showed that the MTT assay was not the best cell enumeration assay when considering a number of parameters that must be weighed up when a cell enumeration assay is selected: the linear range of the MTT assay showed the highest variability which suggests compromised accuracy; it showed high variation in the calculated IC_50_ concentrations of the glycolysis inhibitors after a set number of experiments; interference between the MTT assay and all of the tested glycolysis inhibitors were observed. This study has highlighted that there can be problems with the MTT assay which should be noted. Cell enumeration assays to be used to assess new potential drugs or combinations should be assessed for potential interference by the compounds and suitability determined prior starting the evaluation.

Overall, the SRB assay performed best when all test parameters were considered, suggesting that this assay would be the most time- and cost-effective assay for preliminary cytotoxicity screening. It is therefore recommended that the SRB assay replace the MTT assay as the cell enumeration assay of choice in preclinical testing of potential therapeutic agents having oxido-reductive potential.

## Additional files

Additional file 1:
**The IC**_**50**_ **concentrations of 3-bromopyruvate calculated for MCF-7 cells after 24 or 72 hour incubation calculated per experimental repeat.** Results are indicated as IC_50_ ± SEM. Where no values are given these could not be calculated as the assay reported relative cell survival of greater than 50% at the tested concentrations (n = 4). Additional file 2:
**Interference of three glycolysis inhibitors with the NRU, RES and SRB assays in cell-free systems (n = 3).**

## Abbreviations

2DG: 2-deoxyglucose
3-BrPA: 3-bromopyruvate
ATP: Adenosine triphosphate
DMSO: Dimethyl sulfoxide
FCS: Foetal calf serum
IC_50_: 50% inhibitory concentration
LON: Lonidamine
MTT: 3-(4,5-dimethylthiazol-2-yl)-2,5-diphenyl-2H-tetrazolium bromide
NADH: Nicotinamide Adenine Dinucleotide
NCI: National Cancer Institute of the United States of America
NRU: Neutral red uptake assay
PBS: Phosphate buffered saline
RES: Resazurin reduction assay
SEM: Standard error of the mean
SRB: Sulforhodamine B assay
USA: United States of America
