# Supplementary figures and images for: Limitations of the 3-(4,5-dimethylthiazol-2-yl)-2,5-diphenyl-2H-tetrazolium bromide (MTT) assay when compared to three commonly used cell enumeration assays
Source: BMC Res Notes. 2015 Feb 20;8:47. doi: 10.1186/s13104-015-1000-8 (PMC4349615; doi:10.1186/s13104-015-1000-8)

3-BrPA

2-DG

LON

NRU Assay

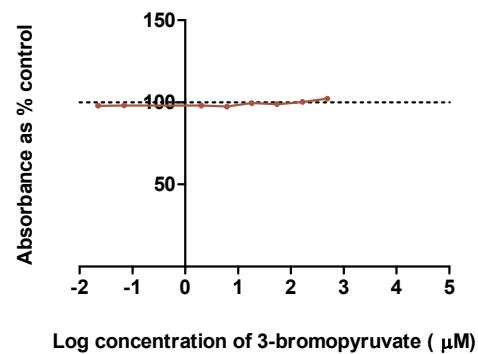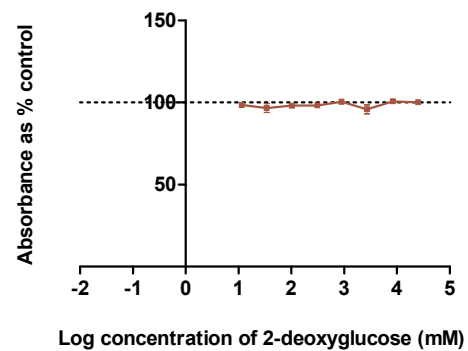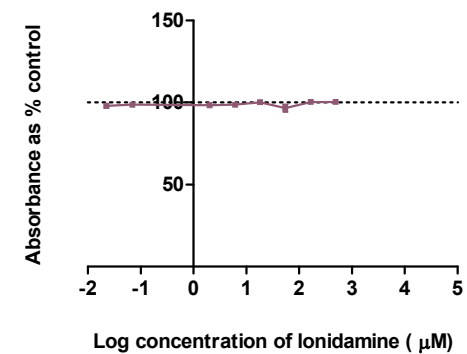

RES Assay

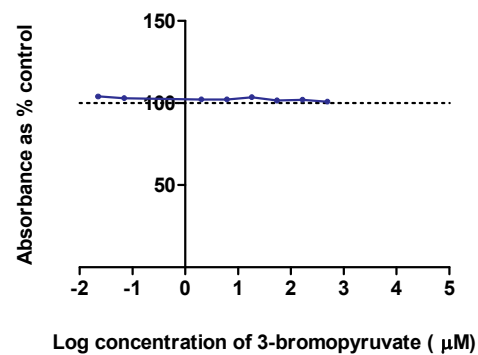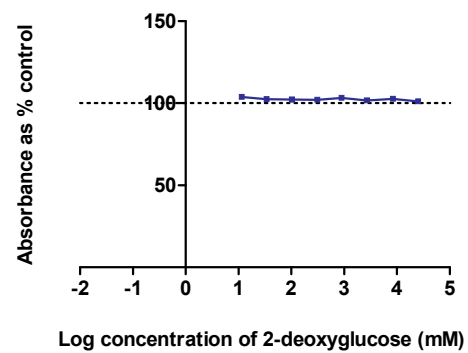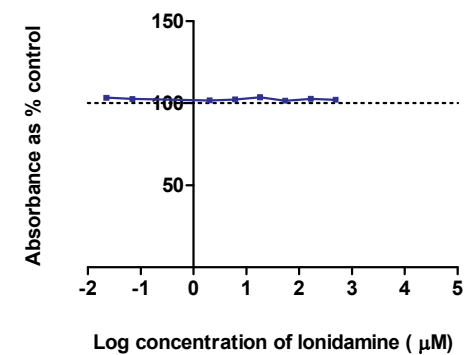

SRB Assay

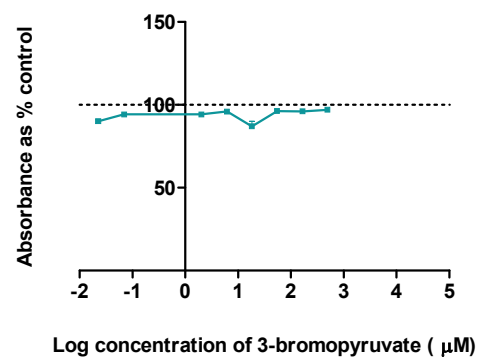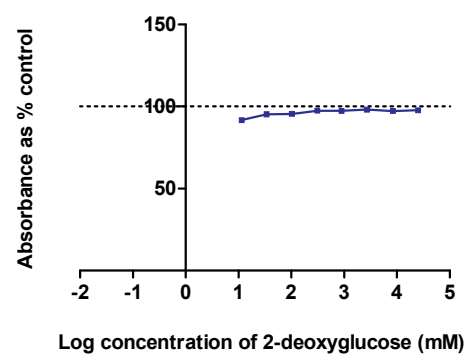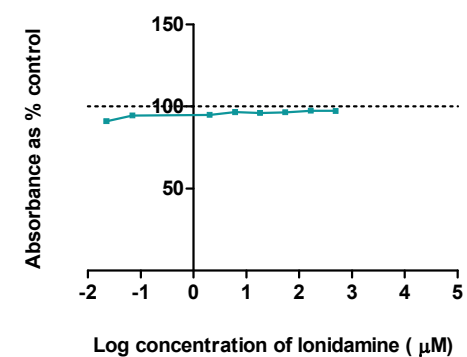

Supplement: Additional file 2: — Interference of three glycolysis inhibitors with the NRU, RES and SRB assays in cell-free systems (n = 3). [file 13104_2015_1000_MOESM2_ESM.pdf]
